# Elements of Materia Medica and Therapeutics

**Published:** 1845-03

**Authors:** 


Art. IV.—*Elements of Materia Medica and Therapeutics.* By John P. Harrison, M.D., Professor of Materia Medica and Therapeutics in the Medical College of Ohio. Vol. I. Cincinnati: Desilver & Burr. 1845. 8vo. pp. 405.

The author of this work expresses, in his preface, a hope that it will be found useful in the following particulars: "1st, by an ample elucidation of topics connected with Therapeutics—such, for example, as Indications of Cure, and other kindred points. 2d, in an abridgment of the cumbrous catalogue of medicines, by the expurgation of many inferior and effete articles. And 3d, by a particular explication of the medicinal powers of our most effective means of cure." He therefore comes before the profession as a reformer. He has a hypothesis to defend, and a new arrangement of the *materia medica* to propose. The paramount object of his work, he says, is utility.

It is our intention to look somewhat critically into the claims of this work, and to determine, if we can, how far the object of its author has been attained. This will require an examination of its doctrines, its practical teachings, and its style. If these prove to be good; there will be no longer any question as to the influence which the work is calculated to exert upon the profession, and we shall take pleasure in bearing our humble testimony to its merits. If, on the contrary, any substantial ground of objection be shown either to the style in which the work is written, or to the scope of its doctrines, this must be held to abate, so far, its claims to the favor of the profession. We are ready to welcome every valuable accession to medical literature, and all the more cordially if its auther chance to be a western man, and a neighbor, for we will not deny that we have our sectional partialities; but there can be no apology, at this day, for putting forth a work of inferior merit on Materia Medica. The numerous excellent treatises on this branch of medicine, which have been published within a few years, must be admitted to preclude any new system not coming highly recommended by its matter, its arrangement, or the style and manner of its execution. How far the work before us can lay claim to such recommendation, it is our purpose now very candidly to inquire.

At the threshold, the author gives his readers to understand that he has endeavored to place the question of the *modus operandi* of medicines on a footing 'exclusively vital'—assuming an 'apparently hostile attitude towards the very popular doctrines of humoralism.' These doctrines, he admits, 'are venerable from their antiquity, and respectable from their universality.' He adds, that 'coming down from the Coan sage, they have, with hereditary authority, been received by the profession as the most, if not only correct, explication of the mysteries connected with the agency of foreign bodies upon the system.' His inference on this point is, that—

"If true, the humoral doctrine, so universally acceptable to the people, and of such ready capability of easy application, should unhesitatingly be adopted, and transferred to the bedside. If medicines act by their introduction into the blood, then we may, and should, make a quick appeal to such a truth, not merely to help us out of our *theoretical perplexities*, but it should be made available to our methodus medendi.

"Happy would it be for mankind, and most auspicious would its bearings be on the art of healing, could we make the doctrines of the humoral pathology our 'guide, philosopher and friend.' By opening a vein and pouring in our medication upon the blood, disease would soon be rebuked. By ascertaining the morbid matters in the blood, and directly neutralizing them by injections into the current of the circulation, a direct attack would be made on the very *germinal elements* of the disease, and thus a rapid destruction of it ensue."

Still, our author does not deny that medicines find their way into the circulation. On the contrary, he allows that they 'do enter the blood, and are to be detected in the secretions.' This would appear to be a frank and unreserved surrender of the question, but not so meant the author to have it understood, for in another place we find him employing the following language:

"If medicines pass into the circulation they become endowed, like all digested substances, with the attribute of assimilation, and are no longer foreign bodies, but integral parts of the circulating fluid. As integral parts of the circulating mass, they are no longer under the laws which determined their mode of action on the body when out of the blood, but in common with the other heterogeneous constituents of that compound fluid, can exercise no controlling interference on the functions of life."

So again:

"In virtue of the power of digestion and assimilation, foreign substances are obliged to undergo a special process before they are admitted into the blood.

"In consequence of this great law of vitality, no substances alien and hurtful to the system are allowed to penetrate, and diffuse their noxious power, beyond the organs whose duty it is to watch over and guard the organism from the introduction of unassimilated materials into the circulation."

And again:

"When madder colors the bones, or nitrate of silver the skin, or rhubarb tinctures the urine, these substances having been assimilated to the organic tissues by passing into the blood, no longer are to be regarded as medicinal agents, but as constituents of the blood."

Finally, the conclusion at which he arrives is, that while medicines, in a certain modified form, may reach the circulation, they, nevertheless, 'do not *operate on the blood*, nor do they act on the organism by direct transmission through the circulation.'

The several propositions stated in these quotations are, that no foreign matter enters the circulation; that medicines are assimilated by the digestive process before they can be absorbed, and become then 'constituents of the blood;' that the blood is not affected by medicinal agents; and that these agents never impress the system through the circulation. Unfortunately, they are all unsustained by proof. From beginning to end, they are assertions merely, and the effect of their perusal in every instance, we suspect, will be not only a smile of incredulity, but an expression of surprise, that a very sensible author should set himself gravely about the defence of a dogma so utterly exploded. The propositions are not only without proof, as we have said, but are in the face of all the evidence bearing on the subject. We need not stop to cite the evidence—it is to be found in every book relating to the question, and must be familiar to every student who has read through his first treatise on Materia Medica. He need not have gone further to be supplied with arguments sufficient to overthrow every one of these positions. To us it is strange, that any mind should cling with such tenacity to a doctrine, to which all experience and observation stand opposed. If anything in medical science is settled, it is the fact, that various foreign bodies, medicinal and poisonous, do find their way into the circulation, and, being there, do affect the living economy. *How* they affect it—by what mode of action—is another question, and one not so easily answered. Our author, in reference to this question, uses the following language, which we quote, partly because it is characteristic, but also partly because it indicates an error into which he has himself fallen. He says:

"The restless and eager spirit of speculation, unsatisfied with a careful generalization of the facts treasured up by a minute scrutiny of the modus operandi of a medicine, desires to penetrate beyond the sensible phenomena, and to unveil the secret workings of nature in the case. We wish not to discourage this original tendency of the human mind for abstruse enquiry into the causes of things; but in all philosophical pursuits it is absolutely essential that we possess some restraining power over this restless desire to penetrate the mysteries of nature, lest it degenerate into a wayward mood of mind, which, spurning the sober bounds of rational investigation, sports with the gay creations of fancy as substantial and demonstrable realities."

Not content with the simple fact, that medicines are found in the secretions and in the blood—that when that fluid has degenerated from its natural state, and has become deficient in red globules, iron restores it to a healthy condition—he has 'spurned the sober bounds of rational investigation, and sports with the gay creations of fancy'—the fancy, that iodine, oxalic acid, chlorine, nitrate of silver, and arsenic, when they are taken into the system cease to be foreign matters, and become '*constituents of the blood!*' It is, indeed, pure fancy. No fact or analogy in nature lends countenance to it. Not only are the substances not assimilated, but they continue to exert their peculiar influence after they have gained admission into the circulation. Whether they do this by their action upon the brain or other vital organs, or by affecting the nerves of the veins in which they circulate, or by some other explicable or inexplicable mode, is a question upon which the minds of physiologists are still unsettled. This may be one of those 'secret workings of nature,' which it is not permitted us ever to 'unveil.' But let us not deny what our senses teach us, because we cannot comprehend what goes on in the animal economy beyond the reach of our vision. Let us not deceive ourselves and others by terms which have no meaning, nor fancy that we have explained any process when we have put it upon a footing '*exclusively vital.*' Above all, let us be cautious, in works intended for beginners, as in all cases where we are liable to lead others astray, not to assume that as science which has no foundation but conjecture, and which all our knowledge contradicts.

This labored effort of our author to revive an obselete idea, must be regarded by his readers, with now and then a rare exception, as a waste of time, and a sad misapplication of ingenuity. It occupies more than thirty pages of the volume, and makes one of the least gracious introductions to it that could well be imagined. In future editions we shall be glad on the author's own account, not less than for the sake of true science, if we shall find that it has been suppressed. 'Speculative views on the modus operandi of remedies,' he says, in his own peculiar way, 'are apt to *merge* into the *illimitable*, and become too refined and attenuated for practical purposes;' and most surely is the remark true of his speculations, on which account we are not sorry to take leave of them, and proceed to other portions of the work where we shall find more to approve.

The classification of remedies has always been a troublesome point with writers on Materia Medica. Under the four heads of '*evacuants*, *excitants*, *secretants*, and *narcotics*,' Professor Harrison thinks all remedial agents might be arranged. But he prefers, to quote his own words, 'a collocation rather more extended, and which more completely quadrates with our six governing therapeutical indications.' Consequently, to the list just given, he adds two other classes—'*alterants* and *derivatives*, or revulsives.' We agree with him, that 'simplicity is a very desirable characteristic in all scientific matters,' and shall not find fault with his classification on that ground. No doubt it will be criticised, for nearly every writer has his favorite arrangement. We have none, and that of Professor H. strikes us as combining as many advantages as most of the others.

On another point we have more difficulty. Our author says:

"The hand of judicious reform should expurgate the cumbrous list found in our works on Materia Medica."

Then, referring to the multitude of medicinal agents enumerated by Pereira, Dunglison, Eberle, Thompson, and others, he goes on in the following strain, which will be likely to recall to the reader's mind the remarks of the author about the desirableness of 'simplicity in scientific matters:'

"Is it not very apparent that such redundancy is both unscientific and injurious? Derogating from that simplicity and directness which should ever characterize all our researches into the sanative efficiency of the articles employed in the practice of medicine, and by its encumbering weight impeding the march of enlightened therapeutics."

All this may be very true; but what hand is to be trusted with the pruning-knife? Who is to decide how far the work of reform shall be carried, what articles shall be lopped off, and what left? A favorite medicine of one author might be thrown out as inert by another. Professor H. would proscribe one agent as useless, and, in turn, might find a valued remedy consigned by Thompson or Dunglison 'to the tomb of all the Capulets.' We cannot foresee where this would end, and, therefore, while we agree that in elementary works it may be profitable to abridge the list of medicinal substances, we incline to the retention of the entire list in the larger systems.

We have said that we are not inclined to make objections to the classification of our author, but, at the same time, we are not prepared to unite with him in his condemnation of other systems; as thus—

"What a sad *dereliction* from that precise and perspicuous collocation, or distribution, which should obtain, is exhibited in those tables of classification, where remedies are arranged under the respective heads of vital agents, chemical agents, and mechanical agents."

Or as in the following sentence, referring to the same:

"All this appears to our mind very much like the old scholastic subtlety—a curious piece of mosaic refinement, entirely at variance with that *axiom already announced*, that all medicines act upon the powers of life, and not in virtue of any chemical law."

The *axiom* already announced? An axiom is an established principle in science or art—a self-evident truth. And is it indeed so fully settled, that no medicines act 'in virtue of any chemical law?' Is it an established principle, that antacids and anti-lithics have no such action? Is it perfectly clear at first sight—so evident that no process of reasoning can make it plainer—that iron in cases of anæmia has nothing chemical in its action, that the influence of anthelmintics is purely vital, and that baths, pustulants, and cauterizing agents involve no chemistry in their operation? If so, it is strange that writers on this subject have not been able to see it, or, to speak more correctly, have so universally continued to deny it. That is a queer sort of an '*axiom*,' it must be confessed, which nearly every body agrees in rejecting.

The chapter on "*the circumstances which modify our therapeutical indications*," contains many just remarks, and evinces study and observation. We do not, however, perceive any particular force in the objections of the author to the theory of specifics, upon which, he says, 'the older writers dwell with fond and pertinacious speculation.' No one, we presume, believes in this 'supposititious *specifica qualitativa* of medicinal agents,' to give his own words again, a remedy which, under all circumstances, 'will so act on the pathological entity, or germinal matter of the disease' as to restore health. In this sense there is no specific in medicine, nor in anything else. Our remedies, all things being the same, always produce the same effect; and that is as much as can be affirmed of the operation of any agent in chemistry or physics. Quinine, mercury, and vaccinia are as truly entitled to the term specific, as are alcohol and nitric acid. Quinine generally arrests chills; mercury cures syphilis; and vaccination prevents small-pox. It would, no doubt, be correct to say that they will always do so under the same circumstances. But they occasionally fail to produce these effects. And the same may be said of alcohol and nitric acid. Alcohol, among many remarkable properties, has the effect of hardening the brain, and for that purpose is employed by the anatomist; but now and then it fails. When the brain has suffered what is called, technically, *ramollissement*, it is found that alcohol does not harden it. So of nitric acid: under favorable circumstances—always, when there is nothing to oppose the union—it may be made to unite with soda and form a salt. But, here as in the other cases, to insure the result we must have certain conditions. If the soda be combined with sulphuric acid, nitric acid will not combine with it. We repeat, therefore, that the argument of our author against specifics does not appear to us to possess any special validity. The only difference that we can see between him and others is as to the meaning of the term. As he defines it, there is no specific; but as commonly understood, most medicines may be said to have something specific in their action.

Since, then, we are unable to perceive any marked peculiarity in the views of our author on this point, we are not prepared to concede to him the superiority of position, which he seems to claim in the following remarkable sentence:

> "In the fulfilment of our six indications of treatment, already discussed, we can, in pursuance of a just scientific methodus medendi, prosecute plans of therapeutic procedure, susceptible of those variations required by the endless mutabilities of disease, whilst at the same time we decline reliance upon dubious and intangible speculations to guide our course."

As we have quoted one sentence, the peculiarities of which can hardly fail to strike the reader, we are tempted to quote a few others, of kindred character, which we find in the discussion of a subject closely allied to the last. The author is treating of the *indications of cure*, when he breaks out in the following strain:

"Then why, with fond and *illusive earnestness*, *speculate* about the *supposititious*, *inherent*, *independent vital endowment of the blood?* Why, with a *fervid fancy*, *heat our minds in the chase of a nonentity?*

"How far can the humoral pathology assist us, in the detection of the true indications of cure in disease. Does this antique, venerable and much venerated system of pathology, throw no light on the true therapeutical indications by which scientific medicine is to be governed at the bed-side? Here is the field on which this time-hallowed and hoary-headed theory, or rather hypothesis, should achieve its most signal triumphs. But alas for its advocates! It deserts them at the bed-side; and at the very moment when its illustrations and enforcement are most anxiously anticipated, like the fabled hero of the Æneid, it escapes in a cloud from the scene of action, and leaves its votary to seek for other aids and better lights to guide him."

We would simply remark, concerning the doctrine of these passages, that there is a wide difference of opinion between Professor Harrison and Professor Andral on this subject, and that the weight of testimony compels us to take sides against our countryman. Prof. H. asserts that—

"There is not a single indication of cure, that we can build on these supposed morbid states of the blood."

Professor Andral, on the other hand, insists that much may be inferred from the appearances of the blood in disease, and adduces so much evidence in favor of that opinion, as was shown in a former number, that most readers, we suspect, will be strongly disposed to give it some credit.

Occasionally, our author manifests a disposition to take leave of these 'dubious and intangible speculations,' which, he owns, 'are apt to merge into the illimitable,' and to confine himself to the more obvious effects of medicines, as having 'stronger claims on our attention than the more fascinating employment of indulging in theoretical explanations of the occult modes of their action.' But he does not continue long in this vein. The strong propensity returns, and hardly has he confessed the fruitlessness of such inquiries, before we find him busy again 'explaining the inexplicable.' The ultimate action of remedies, at one moment, appears to him 'occult'—concealed by an impenetrable veil; but the next he is ready to pronounce positively upon its character, as if he had been admitted behind the curtains and understood all:

"Medicines operate in virtue of a dynamic, and not from any chemical or mechanical mode of action."

And so, again, of the manner in which certain agents operate upon the blood, and restore it to a healthy condition:

"If the blood is thus purified," (by diet and mercurials) "it is *consecutive* upon a prior *rectification* of *disordered function* in the *life of nutrition*."

The following are much in the same style, and if they fail to convey any clear idea to the mind, it cannot be attributed to any lack of words:

"Primordially, the action of an alterant is upon the vital properties of the system, in virtue of the relation which exists between the state of those vital properties, and the article administered."

"From deductions, drawn from a wide field of experience in the profession, we are warranted in referring the *peculiar* power of alterant medicines to a *special medication* which they *create*."

"Incidental, or consequential, to the power possessed by alterants in so modifying the morbid conditions of the organs, as to subvert anormal processes going on in them, there are various evidences of a more energetic and harmonious movement established in the vital endowment of the organic structures. These evidences of a renewal of the healthy state of the parts originally diseased, or of the sympathizing portions of the frame, should be placed among the collateral and resulting phenomena, which accompany, or spring from, the primary alterant action."

The author gives sound advice to young physicians in his chapter on *the theory and art of prescribing*. In the West, especially, he is persuaded, we are prone to attempt too much by our 'heroic remedies,' and in that view, 'most importunately does he solicit the young physician to beware of an intemperate and reckless earnestness in the prosecution of his schemes of cure.' He continues, in his own peculiar way:

"Polypharmacy is bad enough; so is imprecision in the giving of medicines; but a too zealous and eager spirit of dosing, of multiplying dose upon dose, even when done with a precise, accurate, and clear view of the diagnosis, *may inflict more irreparable amount of mischief* upon the *patient's welfare*, than can be compensated for by the *adroitness* and *fine tactics* by which formulæ may be constructed."

On this point, and on all practical subjects, the views of our author strike us as, in the main, sound and judicious, and our impression of the volume before us is, that it gives advice which the young practitioner may safely follow at the bedside. However the mind of the author may be warped by certain theoretical notions, when he comes to speak of the application of remedies to disease, he seems to be free from unwise prepossessions, and ready to adopt whatever experience may have sanctioned in all the systems. The practice he recommends is what would be styled energetic, or bold, rather than expectant. There are points in it to which we might object; but as a whole, we have no doubt, it will be approved by those who have had most experience in the management of our diseases.

After all, is not that the principal matter? The great aim of a book on Therapeutics, is the establishment of a safe and successful plan of practice, and that being attained, we may pass the speculative opinions of the author by, as of secondary importance. Still these have their consequence, and must be taken into account in the general estimate of the work. So, also, must be the style in which the matter is conveyed. The volume before us if tried by the first of these tests will pass well; but if subjected to the latter, our conviction is, that an unfavorable sentence must be passed upon it.

Of the cardinal doctrine of the work, which pervades every portion of it, and appears to hold the mind of the author as by a sort of fascination, it is sufficient to say, that it is obsolete. No galvanizing process can ever excite it to more than a few mimic movements, which no one is in any danger of mistaking for those of life. It is in its grave, 'with forty mortal murders on its head,' and there, 'after life's fitful fever,' it should be allowed to rest.

We shall not attempt to characterize the style of our author. Wherever, in this article, we could do so, we have communicated his views in his own language, and have thus placed before the reader the materials for forming an opinion touching this point. Such specimens might be multiplied to almost any extent, for they are afforded by every page, and nearly every paragraph of the book. Nothing seems so much to the author's taste as what he would style *verba sesquipedalia.* Simplicity and brevity of expression he studiously shuns, as something beneath the dignity of a scholar. He delights in stringing together the longest words, and in piling epithet upon epithet, until his sentences might be said to *groan under them.* Thus in his lively description of the follies of Homœopathy he says:

"Proceeding according to his plan of exiguity, or attenuation, and impregnating each reduction with fresh medical virtues by his rubbing or shaking, one grain of physic will finally answer to dose every man, woman, and child on the face of the earth, all their lives, till, in the ordinary course of mortality, they are all carried to the grave."

It was not enough to say that it would dose them '*all their lives,*' but he must add—'*till*, in the ordinary course of mortality, *they are all carried to the grave.*' Instead of vital, he has the action of medicines to be 'dynamic;' and for a table of doses, he has a 'posological table.' His sedentary persons 'bear with less tolerance,' and his pupils are to engage in

'clinical perquisition,' and be on their guard against 'speculative plausibilities' which 'merge into the illimitable.'

Such sentences as the following are not unfair specimens of the manner in which the author delights to impart instruction:

"Modification of action is induced by medicinal combination, in the creation of a new therapeutic result."

"Can we imagine that this infinitesimal dose will operate any change on each molecule of the blood; or that the visions of homœopathic power will be realized in each texture of the body?"

"Calomel given alone is a remedy of great capability of remedial power, but the chief curative results which arise upon the exhibition of this effective weapon of medical aggression, are seen subsequent to a combination of the article with antimony, or opium, or a union of all three."

"Where ætal and scrofulous predispositions are conjoined with this engrafted, or superadded disturbance of the chylopoietic viscera, there is frequently engendered incurable lesions of secretion, or structure, in the encephalon or thorax."

'Organs giving egress to the peculiar fluids of their secretory elimination,' 'emunctorial outlets,' 'the parturient uterus stimulated to intense expulsive contractions,' 'transmitted pravity of constitution,' 'beyond the warrantable bounds of a sound induction,' 'cutaneous phlogosis,' 'a better hæmatosis, 'modifications imparted to the molecular movements of the blood in the terminal branches of the vascular system:'—these and numberless similar expressions occur continually in the work. We suppose that in quoting them, after the numerous extracts already given, we afford the reader the fairest means of judging of the merits of its style. They will probably start in his mind, as, we confess, they often suggested to ours, the question, whether 'the hand of judicious reform' would not come in well here, to 'expurgate the cumbrous list.' We must say, that of the two—redundancy of things, though they be obscure medicines, and redundancy of words, though of most 'learned length and thundering sound,'—we should be obliged to declare for the former. We are not able to perceive how the pupil is to gain by exchanging matter for phrases—by giving up remedial agents and getting sound in their stead. If the list of remedies is to be retrenched, he is not unreasonable in asking that the language in which they are described be also confined within reasonable bounds. Poverty of matter and exuberance of style might reasonably be expected to provoke his complaints. Y.

